# Fast Screening of Protein Tyrosine Phosphatase 1B Inhibitor from *Salvia miltiorrhiza* Bge by Cell Display-Based Ligand Fishing

**DOI:** 10.3390/molecules27227896

**Published:** 2022-11-15

**Authors:** Xiaolin Bai, Wenqin Fan, Yingjie Luo, Yipei Liu, Yongmei Zhang, Xun Liao

**Affiliations:** 1 Chinese Academy of Sciences, Chengdu Institute of Biology, Chengdu 610041, China; 2 University of Chinese Academy of Sciences, Beijing 100049, China; 3 Department of Molecular Science, The University of Western Australia, Perth, WA 6000, Australia; 4 Polus International College, Chengdu 610103, China

**Keywords:** immobilized PTP1B, drug screening, cell surface display, salvianolic acid B, traditional Chinese medicine

## Abstract

*Salvia miltiorrhiza* Bge is a medicinal plant (Chinese name “Danshen”) widely used for the treatment of hyperglycemia in traditional Chinese medicine. Protein tyrosine phosphatase 1B (PTP1B) has been recognized as a potential target for insulin sensitizing for the treatment of diabetes. In this work, PTP1B was displayed at the surface of *E. coli* cells (EC-PTP1B) to be used as a bait for fishing of the enzyme’s inhibitors present in the aqueous extract of *S. miltiorrhiza*. Salvianolic acid B, a polyphenolic compound, was fished out by EC-PTP1B, which was found to inhibit PTP1B with an IC_50_ value of 23.35 µM. The inhibitory mechanism of salvianolic acid B was further investigated by enzyme kinetic experiments and molecular docking, indicating salvianolic acid B was a non-competitive inhibitor for PTP1B (with Ki and Kis values of 31.71 µM and 20.08 µM, respectively) and its binding energy was −7.89 kcal/mol. It is interesting that in the comparative work using a traditional ligand fishing bait of PTP1B-immobilized magnetic nanoparticles (MNPs-PTP1B), no ligands were extracted at all. This study not only discovered a new PTP1B inhibitor from *S. miltiorrhiza* which is significant to understand the chemical basis for the hypoglycemic activity of this plant, but also indicated the effectiveness of cell display-based ligand fishing in screening of active compounds from complex herbal extracts.

## 1. Introduction

Protein tyrosine phosphatase 1B (PTP1B) is a member of the protein tyrosine phosphatase family, which is mainly localized on the surface of the endoplasmic reticulum [1]. The functional balance of PTP1B and protein tyrosine kinases (PTKs) is crucial to regulate the phosphorylation of tyrosine [2]. Excessive accumulation of PTP1B causes various diseases including type 2 diabetes, obesity, and breast cancer [3,4,5]. It is a negative modulator of insulin signaling and has been recognized as a potential target for insulin sensitizing for the treatment of diabetes. Up till now, several PTP1B inhibitors have been reported as leading compounds for the above-mentioned diseases, but most of them failed in clinical trials because of poor clinical efficacy, severe side effects, and drastic weight loss [6]. Therefore, it is of great significance to discover novel PTP1B inhibitors for development of new drugs.

Traditional Chinese medicine (TCM) has been used to fight diseases for thousands of years, and is a precious pool for discovery and development of new drugs. *Salvia miltiorrhiza* Bge is a famous medicinal plant used in TCM for the treatment of a wide variety of diseases such as diabetes, cardiovascular disease, Alzheimer’s disease, and liver disease [7]. Specifically, it is one of the major ingredients in anti-diabetes Chinese herbal formulas [8]. Both *S. miltiorrhiza* and some major compounds in this plant such as cryptotanshinone, tanshinol, and dehydrodanshenol A have been reported to possess a strong inhibitory effect on PTP1B [9,10].

Ligand fishing has been quickly developed over the past decade for screening bio-active natural products. By using the target protein immobilized on certain solid phases as solid-phase extraction adsorbents, active compounds present in complex plant extracts can be specifically extracted via the high affinity interaction between the protein and its ligands [11]. Various solid phases for this purpose have been reported including magnetic nanoparticles (MNPs), halloysite nanotubes (HNTx) [12], hollow fibers [13], cellular membrane [14], and capillary electrophoresis (CE) [15]. While a variety of instrumentation techniques such as high performance liquid chromatography/mass spectrometry (HPLC-MS) [11], matrix-assisted laser desorption ionization time-of-flight mass spectrometry (MALDI-TOF-MS) [16], ultrafiltration liquid chromatography/mass spectrometry (UF-LC-MS) [17], and lab-on-chip were used to identify the fished out active compounds [18]. However, those methods have a common drawback in that the natural conformation of target proteins cannot be maintained during the immobilization process, which usually causes fake results. Very recently, we displayed PTP1B on the surface of *Escherichia coli* cells to obtain a recombinant bacteria (EC-PTP1B) and used it as a new bait for ligand fishing, which exhibited promising potential to overcome such shortcomings [19].

**Figure 1:**
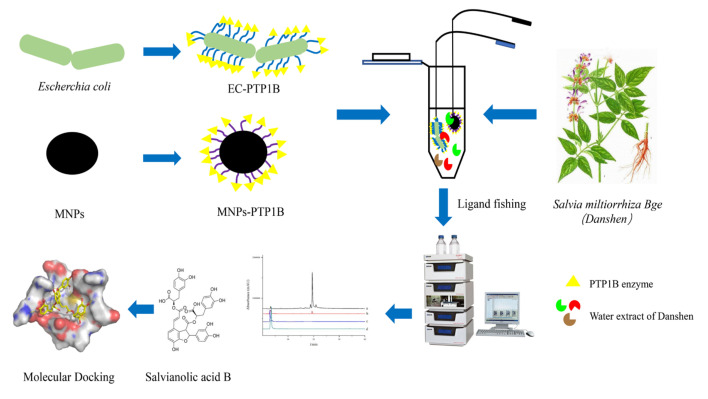
Flow chart of screening PTP1B inhibitors from S*. miltiorrhiza* extract.

In this work, the EC-PTP1B was applied to screen PTP1B inhibitors present in *S. miltiorrhiza* Bge in comparison with the conventional ligand fishing bait of magnetic nanoparticle immobilized PTP1B (MNPs-PTP1B). The stability and enzymatic activity of PTP1B in the forms of free PTP1B, MNPs-PTP1B, and EC-PTP1B were first compared, and the enzyme’s ligand fished out was investigated for its inhibitory mechanism by enzyme kinetics and molecular docking experiments. The schematic illustration of this experimental procedure was shown in Figure 1.

## 2. Results and Discussion

### 2.1. Characterization of the Immobilized-PTP1Bs

**Figure 2:**
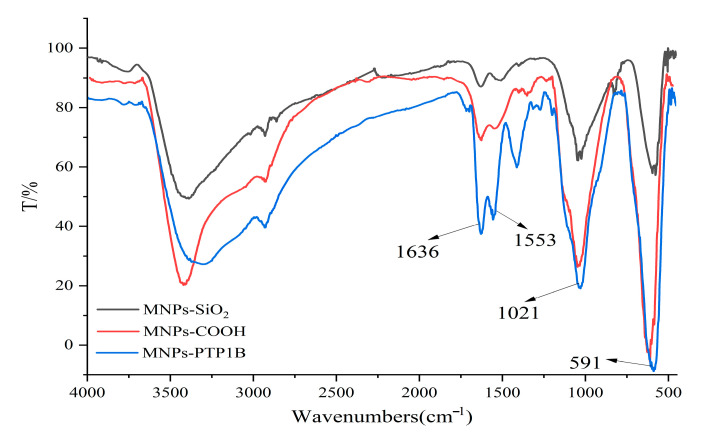
FT−IR spectra of MNPs−SiO_2_, MNPs−COOH, and MNPs−PTP1B.

The PTP1B was immobilized on carboxyl terminated magnetic beads for the first time in this study. FT-IR was used to confirm chemical composition of MNPs-SiO_2_, MNPs-COOH, and MNPs-PTP1B. As shown in Figure 2, the stretching vibration of Fe-O and asymmetric vibration of Si-O-Si of MNPs-SiO_2_ (black line) were observed around 591 cm^−1^ and 1021 cm^−1^, respectively. For MNPs-COOH (red line), the absorption peaks at 1553 cm^−1^ and 1636 cm^−1^ were ascribable to stretching vibration of C = O and bending vibration of N-H, indicating that NH_2_ and COOH groups had been successfully coated on MNPs. In addition, it is obvious that the peaks at 1553 cm^−1^ and 1636 cm^−1^ for MNPs-PTP1B (blue line) were stronger than those for MNPs-COOH, demonstrating that PTP1B was successfully immobilized on the surface of MNPs. The ratio of PTP1B immobilized versus MNPs was about 89 µg/mg measured by Bradford assay.

### 2.2. Effects of pH and Temperature on the Activity of Free PTP1B, MNPs-PTP1B, and EC-PTP1B

**Figure 3:**
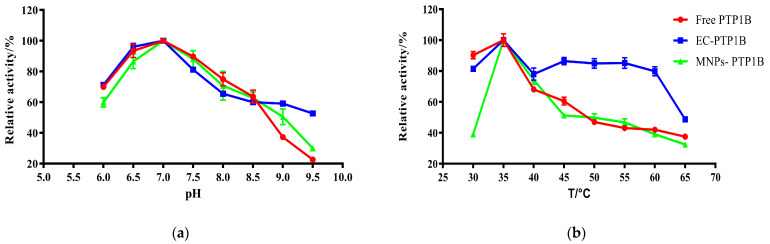
Effect of pH (**b**) and temperature (**b**) on the activity of free PTP1B, MNPs-PTP1B, and displayed PTP1B cells.

The influence of pH on free PTP1B, MNPs-PTP1B, and EC-PTP1B was compared at a temperature of 37 °C in Figure 3a. It was found that the optimum pH values for them were all 7.0. In the pH range of 6.0–7.0, the enzymatic activity of MNPs-PTP1B was weaker than those of free PTP1B and EC-PTP1B, which might be due to the reduction of PTP1B active centers resulting from the covalent immobilization [20]. On the other hand, the MNPs-PTP1B and EC-PTP1B exhibited higher activity than the free one when the pH value was higher than 8.5, indicating that immobilized enzymes are more resistant to extreme pH conditions via restricting changes in enzyme conformation [21].

The effect of temperature (30–65 °C) on the activity of the three types of enzymes is illustrated in Figure 3b. All of them exhibited the highest enzymatic activity at 35 °C, whereas EC-PTP1B was more stable than the other two. It is well known that free enzymes are unstable once added into an in vitro reaction solution. In our case, *E. coli* can provide a natural and intact cell membrane which is similar to the biological environment for the enzyme immobilized onto it [22], leading to the significant higher stability of EC-PTP1B at a high temperature.

### 2.3. Storage Stability of Free PTP1B, MNPs-PTP1B, and EC-PTP1B

**Figure 4:**
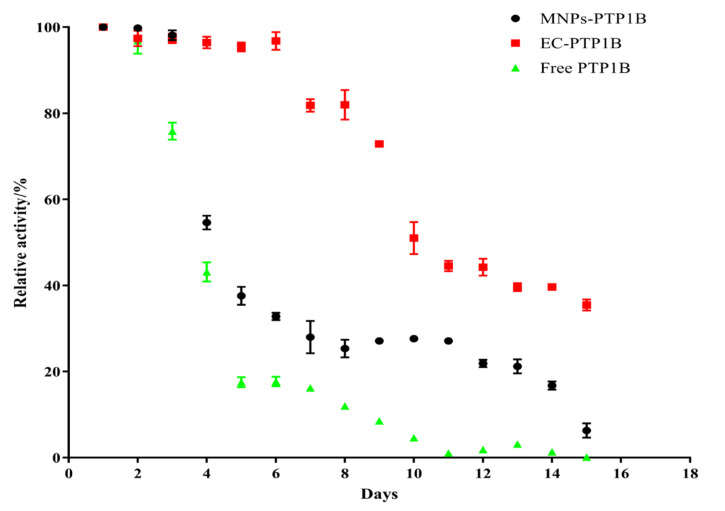
Stability of free PTP1B, MNPs-PTP1B, and EC-PTP1B.

Storage stability is an important parameter for the practical application of the immobilized enzyme. We compared the storage stability of free PTP1B, MNPs-PTP1B, and EC-PTP1B at 4 °C for 15 days. As shown in Figure 4, the activity of EC-PTP1B was maintained during the first six days compared to the sharp drop of the other two. The decrease of activity of all three types of enzymes started from day 7, while EC-PTP1B was still much more stable than the other two by maintaining around 50% of the initial activity until day 10 in comparison to 30% and 5% of MNPs-PTP1B and the free enzyme, respectively. It was reported that the interaction between two proteins which were co-expressed by *E. coli* was much stronger and stable than that between protein and non-biological materials [23,24,25], which might lead to the excellent storage stability of EC-PTP1B. Further, MNPs-PTP1B exhibited higher storage stability than the free PTP1B, which might result from the covalent bonding between the MNPs and the enzyme that not only maintained the conformational stability of enzyme, but also avoided the undesirable aggregation of the free enzyme [26].

### 2.4. Ligand Fishing from the Standard Mixture by MNPs-PTP1B and EC-PTP1B

**Figure 5:**
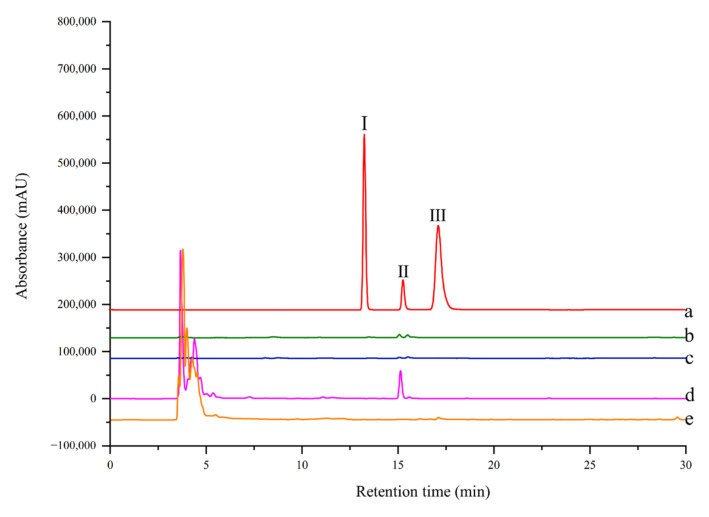
HPLC chromatogram from a model mixture before and after ligand fishing (a) the model mixture: two non-binders (0.05 mg/mL 4-hydroxycinnamic (Ι) and 0.05 mg/mL coumarin (III)) and one binder (0.05 mg/mL rutin (II)). (b) the compounds obtained by ligand fishing using MNPs-PTP1B. (c) the compounds obtained by ligand fishing using MNPs. (d) the compounds obtained by ligand fishing using EC-PTP1B. (e) the compounds obtained by ligand fishing using control cells (*E. coli* cells expressing only ice nuclein protein).

The selectivity of ligand fishing by EC-PTP1B and MNPs-PTP1B was compared using the standard mixture containing one ligand (rutin) and two non-ligands (4-hydroxycinnamic and coumarin) of the enzyme. As shown in Figure 5, only EC-PTP1B extracted the positive compound rutin, indicating that EC-PTP1B was efficient for fishing the enzyme ligand. MNPs-PTP1B as a traditional ligand fishing bait was supposed to fish out rutin, however, it failed this time. It might be explained by the destruction of the natural conformation and active centers of PTP1B resulting from the covalent binding of the enzyme to MNPs.

### 2.5. Ligand Fish and Analysis of Aqueous Extract of S. miltiorrhiza

**Figure 6:**
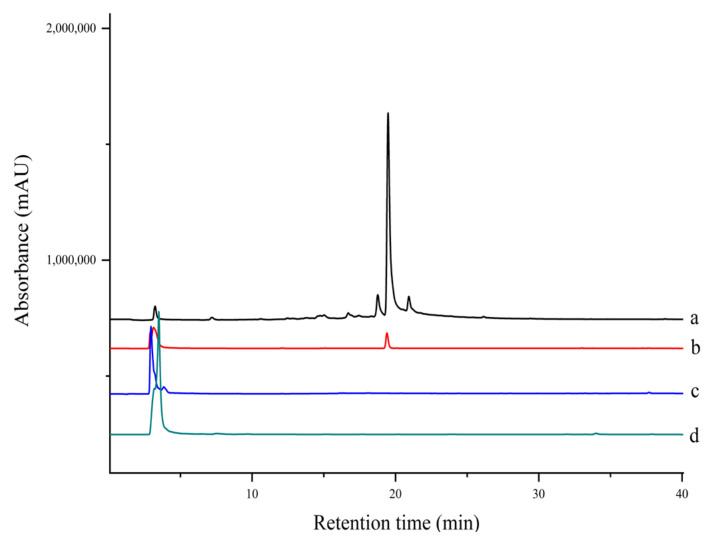
HPLC chromatograms of extract and ligand fishing of (a) *S. miltiorrhiza* aqueous extract, (b) ligand fishing by EC- PTP1B, (c) ligand fishing by the control cells (*E. coli* cells expressing only ice nuclein protein), and (d) ligand fishing by MNPs-PTP1B.

Several PTP1B inhibitors have been reported from methanol extract of *S. miltiorrhiza*, such as grandifolia F, ferruginol, tanshinone IIA, tanshinol B, and isocryptotanshinone [9,27]. However, there is no report on the aqueous extract of this plant. Since traditional herbs are basically consumed in the form of decoction with water; the aqueous extract is supposed to be more important as far as pharmacological significance is concerned. As shown in Figure 6, there was one compound fished out by EC-PTP1B, but none was fished out by MNPs-PTP1B. It is the same interesting result as in Section 2.4. As mentioned above, this may be explained by the natural conformation as well as active centers of protein displayed at the surface of *E. coli* being well preserved, making it capable of interacting with its ligands in the aqueous extract. In contrast, the covalent binding of PTP1B to the MNPs probably destroyed the two factors, resulting in the unsuccessful fishing of the enzyme ligand.

The compound fished out by EC-PTP1B was identified as salvianolic acid B using HPLC-MS/MS and NMR in Supplementary Materials (Table S1 [28] and Figure S1, Figure S2 and Figure S3). The molecular formula of C_36_H_30_O_16_ was deduced by HRMS (*m*/*z* 718.1534 [M + Na]^+^, calcd. for 741.1432), and its retention time was identical to the standard compound.

### 2.6. Inhibitory Mechanism of Salvianolic Acid B against PTP1B

**Figure 7:**
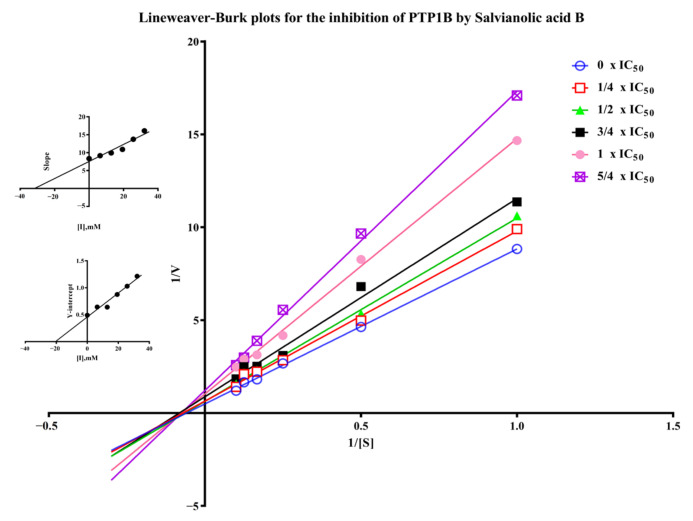
Lineweaver-Burk plots for the inhibition of PTP1B by salvianolic acid B. (Insets) Replots of the slopes and Y-intercept of the Lineweaver−Burk plots.

Salvianolic acid B is one of the major polyphenolic ingredients in *S. miltiorrhiza* possessing various biological activities such as neuroprotection, anti-inflammatory, antithrombotic, and anticancer activity [7,29,30,31]. We found for the first time the PTP1B inhibitory activity of salvianolic acid B with IC_50_ of 23.35 ± 4.48 µM in Supplementary Materials (Figure S4) compared to 9.93 ± 2.74 µM of the positive control sodium orthovanadate [32]. The inhibitory mechanism of salvianolic acid B was investigated by the Lineweaver-Burk plot method. As shown in Figure 7, when the concentration of salvianolic acid B increased, the value of Vmax decreased while Km remained unchanged, suggesting the inhibitor did not interfere with the binding of *p*NPP to the enzyme and it was a non-competitive inhibitor for PTP1B. As a result, Ki and Kis values were 31.71 µM and 20.08 µM for salvianolic acid B, respectively. Recently, various natural compounds were found to possess PTP1B-inhibitory activity, such as shikonin, garcinone E, and kuraridin [33,34,35]. The IC_50_ of them including salvianolic acid B found in this work are between 0 and 50 μM. Their inhibitory mechanism is worth investigating in the future.

### 2.7. Molecular Docking Study

**Figure 8:**
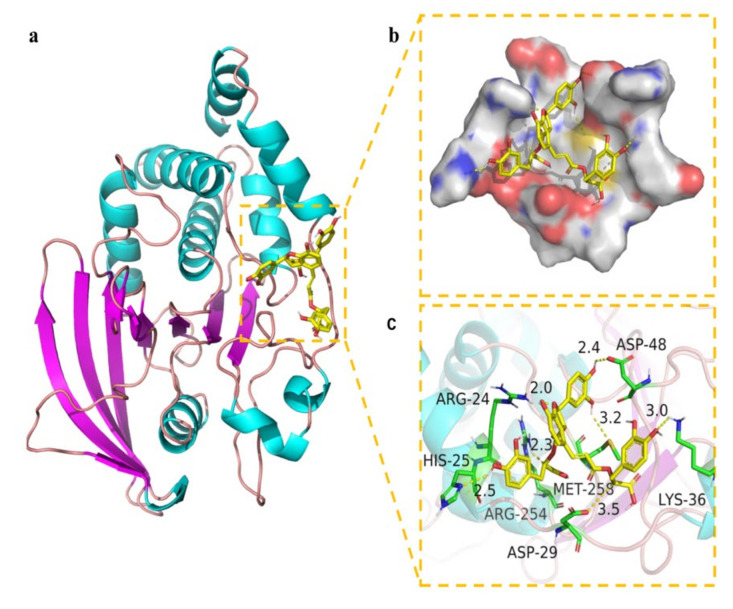
Binding mode of salvianolic acid B to PTP1B. (**a**) The 3D structure of the complex. (**b**) The surface of active site. (**c**) The detail binding mode of the complex. The backbone of protein was rendered in tube and colored in bright blue. Salvianolic acid B compound was rendered in yellow. The yellow dash represents hydrogen bond distance.

Molecular docking is a widely accepted tool for exploring the interaction between drug candidates and proteins in computer-aided drug discovery and design [36]. Pymol 2.1 software was used for this purpose in this work. The results displayed that the binding energy of salvianolic acid B was −7.89 kcal/mol. The 2D and 3D computational binding results between salvianolic acid B and PTP1B are illustrated in Figure 8a,b,c. The rich benzene rings of salvianolic acid B were found to form hydrogen bonds with the amino acid residues of Asp-48, Lys-36, Met-258, Asp-29, Arg-254, His-25, and Arg-24. Based on the above observation, salvianolic acid B exhibited good performance in binding with active centers of the enzyme with a high docking score, suggesting its potential inhibitory effect on the enzyme.

## 3. Materials and Methods

### 3.1. Chemicals and Materials

PTP1B (human, recombinant) was purchased from Sangon Company (Shanghai, China). The rhizomes of *Salvia miltiorrhiza* Bge were generously presented by the Wansheng Agricultural Company of Zhongjiang County (Sichuan, China). A voucher specimen (2019-07) was deposited in the herbarium of Chengdu Institute of Biology, Chinese Academy of Science. Salvianolic acid B was obtained from Lemeitian Medicine (Chengdu, China). Sodium Orthovanadate (Na_3_VO_4_), and para-nitrophenyl phosphate (*p*NPP) were purchased from Macklin Company (Shanghai, China). Tetraethyl orthosilicate (TEOS) and 3-aminopropyltrimethoxysilane (APTMS) were obtained from TCI (Tokyo, Japan). Ferric chloride hexahydrate (FeCl_3_·6H_2_O), Iron(II) chloride tetrahydrate (FeCl_2_·4H_2_O), 4-Morpholineethanesulfonic acid (MES), Hydrochloric acid (HCl), and sodium hydroxide (NaOH) were purchased from Tianjing Kermel Chemical Reagent (Tianjing, China). Acetonitrile and Methanol (HPLC-grade) were obtained from J&K Technology (Beijing, China). Deionized water (18.5 MΩ) was prepared from the Chengdu Youpu Equipment Company (Chengdu, China). The 96-well microtiter plates were purchased from Bioland Technology company (Hangzhou, China).

### 3.2. Apparatus

The High-performance liquid chromatography (HPLC) system includes two LC-20AD pumps (Shimadzu, Japan), an SPD-20A UV-Vis detector, a thermostat column, and an Agilent ZORBAX SB-C18 column (5 µm, 4.6 × 250 mm). The eluation system consists of water with 0.1% formic acid (mobile phase A) and methanol (mobile phase B), and the flow rate was 0.8 mL/min during the following gradient: 0.00–35.00 min, 30–100% mobile phase B; 35.00–40.00 min 100 mobile phase B. The 1D NMR spectra were recorded in CD_3_OD using a Bruker DRX-600 spectrometer (Bruker, Rheinstetten, Germany) with tetramethylsilane (TMS) as the internal standard. Cells were cultured in a constant temperature incubator shaker (Zhicheng Analytical Instrument, Shanghai, China). The ligand fishing process was completed by a high-speed centrifuge (DLABsci Instrument, Beijing, China) and a vortex oscillator (Crystal Instrument, HYQ-3110, USA). HPLC-MS/MS analysis was performed on a Waters ACQUITY system coupled with a XEVO TQ MS triple-quadrupole mass spectrometer (Waters, Milford, PA, USA). A microplate reader (ThermoFisher, Multiskan GO, USA) was used for enzymatic activity assay. A Shimadzu 8030 LC-MS (Shimadzu, Japan) was used for compound identification.

### 3.3. Preparation of PTP1B Immobilized Magnetic Nanoparticles (MNPs-PTP1B)

Firstly, carboxyl terminated magnetic nanoparticles (MNPs) were synthesized according to the same protocol as in our previous work [37,38]. Briefly, 0.7455 g FeCl_2_·4H_2_O and 2.0271 g FeCl_3_·6H_2_O were added in 250 mL ddH_2_O under pH = 9–10 to react for 30 min. The obtained MNPs were coated with a layer of silica using 400 µL TEOS in 150 mL of ethanol for 5 h at 35 °C (pH = 9), which were then modified with amino groups by adding 2 mL APTES in 100 mL 95% ethanol for 24 h at 35 °C. Finally, 0.5 g MNPs-SiO_2_-NH_2_ beads were terminated with carboxyl group by adding 3 g butanedioic anhydride in 30 mL dimethyl formamide for 3 h at room temperature. The above products, i.e., MNPs-SiO_2_, MNPs-SiO_2_-NH_2_, and MNPs-SiO_2_-NH_2_-COOH were characterized by FT-IR. Secondly, PTP1B was covalently immobilized on the carboxyl terminated MNPs as follows. Briefly, 5 mg EDC and 7 mg NHS were used to activate 20 mg of MNPs-COOH beads for 30 min at room temperature, and 0.5 mg/mL PTP1B was incubated with the activated beads in a 5 mL Eppendorf tube (13.7 mM NaCl, 2.7 mM KCl, 10 mM Na_2_HPO_4_·12H_2_O, 1.76 mM KH_2_PO_4_, pH = 7.4) at room temperature over 24 h. The prepared MNPs-PTP1B was suspended in PBS buffer and stored at 4 °C before use. In our study, protein content was measured using bovine serum albumin (BSA) as a standard by the Coomassie brilliant blue G-250 method which was commonly used to quantify the content of protein [39]. The bond PTP1B of MNP was determined by the difference between the initial and residual protein concentrations.

### 3.4. Preparation of PTP1B Displayed Cells (EC-PTP1B)

The *E. coli* cells, which harbored vector pETInaK-N/PTPN1 (as EC-PTP1B) and pMDInaK-N (as control cells), were obtained from our previous study and stored in a storage buffer with 50% glycerol at −20 °C [19]. Both cells were resuscitated and grown in an LB-Kan+ medium (5 g yeast extract, 10 g tryptone, 10 g NaCl, and 50 mg kanamycin dissolved in 1 L ddH_2_O, pH = 7.4) by shaking (200 rpm) at 37 °C until OD_600_ = 0.6. The expression of the PTP1B enzyme on the *E. coli* surface was induced with 0.5 mM isopropyl-β-thiogalactopyranoside (IPTG) at 25 °C for 24 h. After that, displayed cells were washed with PBS buffer and stored in a 50-mL centrifuge tube with 20 mL PBS buffer at 4 °C.

### 3.5. Comparison of the Activity and Stability of the Free and Immobilized PTP1B

The enzymatic activity of PTP1B was assayed using 4-nitrophenyl phosphate (*p*NPP) as a substrate according to a previously reported method with a slight modification [19]. Briefly, the *p*NPP (10 mM, 100 uL) in reaction buffer (25 mM Tris/HCL, 150 mM NaCl, 5 mM MgCl_2_ and 4 mM DTT, pH = 8.5) was mixed with the enzyme solution (100 μL) in an Eppendorf tube incubated at 37 °C for 30 min. The reaction was then terminated by the addition of NaOH (0.1 M, 100 μL), and the product *p*NP was transferred to a 96-well plate to be measured using a microplate reader at 405 nm. All experiments were carried out in triplicate and data shown as mean ± SD.

To compare the enzymatic activity of the three forms of PTP1B, the following five groups of enzyme or control were assayed: (1) free PTP1B enzyme; (2) EC-PTP1B; (3) control cells; (4) MNPs-PTP1B; (5) control MNPs. For MNPs-PTP1B and the control MNPs, 20 mg of each was firstly suspended in 3 mL reaction buffer, from which 100 µL was added to the substrate to start the reaction. After completion of the reaction, the supernatant was collected after magnetic separation and its absorbance was read at 405 nm using a microplate reader. For the other three groups, the free PTP1B was diluted to a concentration of 10 µg/mL, and the EC-PTP1B and control cells were dissolved in reaction buffer to OD_600_ = 0.5 before starting the enzymatic reaction, while the remaining process was the same as described with MNPs-PTP1B.

To compare the stability of the three forms of PTP1B, the effect of various temperatures on the activity of the three forms of PTP1B at pH = 7.5 was first investigated. In the meantime, the influence of pH (ranging from 6.0 to 9.0) on the enzymes’ activity was also evaluated. Secondly, the enzymatic activity of the three forms of PTP1B was tested on 16 consecutive days to evaluate their storage stability.

### 3.6. Validation of Ligand Fishing by MNPs-PTP1B and EC-PTP1B

#### 3.6.1. Preparation of the Standard Mixture

Coumarin and 4-hydroxycinnamic (both are non-PTP1B inhibitors), and rutin (PTP1B inhibitor) were mixed to prepare a standard mixture for validating the selectivity of MNPs-PTP1B and EC-PTP1B for ligand fishing. All the compounds were dissolved in PBS buffer at a concentration of 0.05 mg/mL.

#### 3.6.2. Ligand Fishing from the Standard Mixture by MNPs-PTP1B and EC-PTP1B

MNPs-PTP1B (20 mg) was incubated with 1 mL of the standard mixture at 37 °C for 30 min. After the magnetic separation, the MNPs-PTP1B were washed with 3 mL PBS buffer thrice before 1 mL 50% acetonitrile was added to desorb the ligand. The supernatant after magnetic separation was collected and filtered with a 0.22 µm membrane for HPLC analysis. In the meantime, MNPs were used for ligand fishing following the same procedure as a control for this experiment.

A total of 1 mL EC-PTP1B (OD_600_ = 0.5) was incubated with 1 mL standard mixture at 37 °C for 4 h. Then, the mixture was centrifuged for 15 min (4500 rpm) to remove the supernatant. The EC-PTP1B was washed with PBS buffer thrice before 1 mL of 50% acetonitrile was added to desorb the ligand. After centrifugation, the supernatant containing the ligand was collected and filtered with a 0.22 µm membrane for the following analysis. In parallel, the control cells (OD_600_ = 0.5) were used as bait for ligand fishing following the same procedure.

### 3.7. Ligand Fishing of PTP1B Inhibitor from S. miltiorrhiza

#### 3.7.1. Extraction of *S. miltiorrhiza*

Firstly, 50 g of powdered rhizome of the plant was refluxed twice in 500 mL water in a round bottom flask for 2 h. The aqueous solutions were combined to be concentrated in a rotary evaporator. Because herbs are generally boiled to prepare the decoction and PTP1B of EC-PTP1B or MNP-PTP1B is stable in PBS buffer, the extract was dissolved in PBS buffer to a concentration of 1.0 mg/mL and stored at 4 °C before use.

#### 3.7.2. Ligand Fishing by MNPs-PTP1B and EC-PTP1B

MNPs-PTP1B (20 mg) and EC-PTP1B (OD_600_ = 0.5) were incubated with 1 mL extract of *S. miltiorrhiza* at 37 °C for 30 min and 4 h, respectively. The following steps were the same as described in Section 3.6.2 for the corresponding fishing bait. Similarly, MNPs and EC-PTP1B were used as the control in this experiment.

### 3.8. Inhibitory Assay and Kinetic Study of the Enzyme’s Ligand Salvianolic Acid B

The PTP1B inhibitory activity of salvianolic acid B was tested as described in 2.5 with sodium orthovanadate as a positive control. A series of concentrations of salvianolic acid B (50 μL) was incubated with 50 μL 10 mM *p*NPP at 37 °C for 30 min before 100 μL NaOH was added to end the reaction. The inhibition effect on PTP1B was calculated by the formula: inhibition % = (A_blank control_ − A_sample_)/A_blank control_ × 100%, where A_blank control_ and A_sample_ stands for the absorbance of the blank control and sample.

For the enzyme kinetic study of salvianolic acid B, the inhibition mode and kinetic constants were calculated by the Lineweaver-Burk plot, and six lines were represented by different concentrations of salvianolic acid B (0, 1/4 × IC_50_, 1/2 × IC_50_, 3/4 × IC_50_, 1 × IC_50_, and 5/4 × IC_50_) with a series of increasing concentrations of *p*NPP (1, 2, 4, 6, 8, and 10 mM). The reaction rate was recorded in the first 20 min after the reaction was triggered by *p*NPP.

### 3.9. Molecular Docking Study

Molecular docking was conducted to verify the mode of interaction between the ligand and enzyme. The X-ray crystal structure of PTP1B (PDB ID: 1QXK) was obtained from the RCSB PDB protein data bank, and the resultant structure was processed with the help of Maestro 11.9 software after removing the crystal water, adding a hydrogen atom, repairing incomplete peptide bonds, and minimizing the protein energy. The 3D structure of salvianolic acid B was downloaded from PubChem database. Glide functionalities provided in Schrödinger Maestro software (Schrödinger, Cambridge, MA, USA) were used for the molecular docking [40,41].

## 4. Conclusions

In this study, ligand fishing methods were employed for screening PTP1B inhibitors based on two functional adsorbents, i.e., MNPs-PTP1B and EC-PTP1B. MNPs-PTP1B was synthesized via covalent binding of PTP1B and carboxyl terminated MNPs for the first time. The storage stability as well as the pH and thermo durability of the two adsorbents were investigated. Both were more stable than the free enzyme, while EC-PTP1B exhibited significant improvement over the MNPs-PTP1B. When applied in the ligand fishing of aqueous extract of *S. miltiorrhiza*, it was interesting to find that only EC-PTP1B fished out an active polyphenolic compound of salvianolic acid B, which was found to be a non-competitive inhibitor of PTP1B with IC_50_ of 23.35 µM. This result indicated that enzymes displayed on the surface of *E. coli* is superior to covalently immobilized ones in terms of ligand fishing due to the maintenance of the natural conformation of the enzyme, thus providing a powerful tool for screening active compounds from complex medicinal plant extracts.

## Data Availability

All data are available upon reasonable request.

## Supplementary Materials

The following supporting information can be downloaded at: https://www.mdpi.com/article/10.3390/molecules27227896/s1, Table S1: NMR spectroscopic data (CD_3_OD) for salvianolic acid B; Figure S1: ^13^C NMR (150 MHz, CD_3_OD) spectrum of salvianolic acid B; Figure S2: ^1^H NMR (600 MHz, CD_3_OD) spectrum of salvianolic acid B; Figure S3: MS spectrum of salvianolic acid B (positive ion mode); Figure S4: IC_50_ plot of salvianolic acid B and its chemical structure.
