# Supplementary material for: Fast Screening of Protein Tyrosine Phosphatase 1B Inhibitor from Salvia miltiorrhiza Bge by Cell Display-Based Ligand Fishing
Source: Molecules. 2022 Nov 15;27(22):7896. doi: 10.3390/molecules27227896 (PMC9693971; doi:10.3390/molecules27227896)
Supplement: Supplementary file 1 [file molecules-27-07896-s001.zip › molecules-1997046-supplementary.pdf]

Supporting Information

Fast screening of protein tyrosine phosphatase 1B inhibitor from *Salvia miltiorrhiza* Bge by cell display-based ligand fishing

Xiao-Lin Bai[1, 4], Wen-Qin Fan[1, 4], Ying-Jie Luo[2], Yi-Pei Liu[3], Yong-Mei Zhang[1, *], Xun Liao[1, *]

Affiliation:

1. Chinese Academy of Sciences, Chengdu Institute of Biology, Chengdu, P. R. China
2. University of Western Australia, Perth 6000, Australia
3. Polus International College, Chengdu 610103, China
4. University of Chinese Academy of Sciences, Beijing 100049, China

Corresponding Authors:

Dr. Xun Liao, Email: liaoxun@cib.ac.cn; Tel: +86-28-828290402

Dr. Yong-Mei Zhang, Email: zhangym@cib.ac.cn; Tel: +86-28-82890756

Contents of Supporting Information

Table S1. NMR spectroscopic data (CD_3OD) for salvianolic acid B ………………………...3

Figure S1. ^{13}C NMR (150 MHz, CD_3OD) spectrum of salvianolic acid B……………….......4

Figure S2. ^{1}H NMR (600 MHz, CD_3OD) spectrum of salvianolic acid B…………………….5

Figure S3. MS spectrum of salvianolic acid B (positive ion mode)…………………………...6

Figure S4. IC_{50} plot of salvianolic acid B and its chemical structure…………………………7

Figure S5 The authentication letter of crude drug sample……………………………………..8

Reference………………………………………………………………………………9

Table S1. NMR spectroscopic data (CD_3OD) for salvianolic acid B [1]

	Reference			This work		
position	δc	type	ΔH（*J* in Hz）	δc	type	ΔH（*J* in Hz）
2	87.5	CH	5.87 d (3.9)	88.3	CH	5.84 d (4.8)
3	57.1	CH	4.47 d (4.6)	57.9	CH	4.33 d (4.8)
4	124.6	C		124.7	C	
5	121.6	CH	7.24 d (8.4)	121.7	CH	7.14 d (8.4)
6	118.2	CH	6.90 d (8.4)	118.4	CH	6.82 d (8.4)
7	148.6	C		149.1	C	
8	146.2	C		146.8	C	
9	126.1	C		126.4	C	
α	117.1	αCH=CH	6.28 d (15.9)	117.3	αCH=CH	6.18 d (15.6)
β	142.8	βCH=CH	7.62 d (15.9)	142.6	βCH=CH	7.50 d (15.6)
9	126.1	C		126.4	C	
1'	133.3	C		133.7	C	
2'	117.1	CH	6.83 dd (1.8, 6.3)	117.3	CH	6.72 overlap
3'	116.0	CH	6.80 d (8.1)	116.4	CH	6.75 overlap
4'	144.7	C		146.0	C	
5'	145.5	C		146.1	C	
6'	113.2	CH	6.82 d (1.9)	113.4	CH	6.73 overlap
1''	128.7	C		128.9	C	
2''	116.1	CH	6.67	116.6	CH	6.5
3''	146.0	C		146.6	C	
4''	145.5	C		146.1	C	
5''	116.1	CH	6.65 d (8.0)	116.5	CH	6.52 d (8.0)
6''	121.7	CH	6.45 dd (1.5, 8.0)	122.1	CH	6.28 dd (2.4, 8.4)
7''	37.4	CH2	3.06	37.9	CH2	3.04
8''	73.9	CH	5.21 d (8.7)	74.7	CH	5.16 overlap
9''	171.6	COOH		171.7	COOH	
10''	166.6	C		166.0	C	
1'''	129.1	C		129.3	C	
2'''	116.8	CH	6.71 d (1.7)	116.6	CH	6.63 overlap
3'''	144.6	C		145.1	C	
4'''	144.4	C		145.1	C	
5'''	117.3	CH	6.74 d (8.0)	117.6	CH	6.68 d (7.8)
6'''	121.8	CH	6.70 dd (1.6, 8.6)	122.3	CH	6.60 dd (1.7, 7.8)
7'''	37.1	CH2	3.06	37.5	CH2	2.98
8'''	74.9	CH	5.22 d (9.0)	75.6	CH	5.16 overlap
9'''	170.8	COOH		172.6	COOH	
10'''	171.2	C=O		172.3	C=O	

Note: For the position number, e.g., 1′, 1′′, and 1′′′ et. al., refer to the chemical structure shown in Fig. S4.

Figure S1. ^{13}C NMR (150 MHz, CD_3OD) spectrum of salvianolic acid B

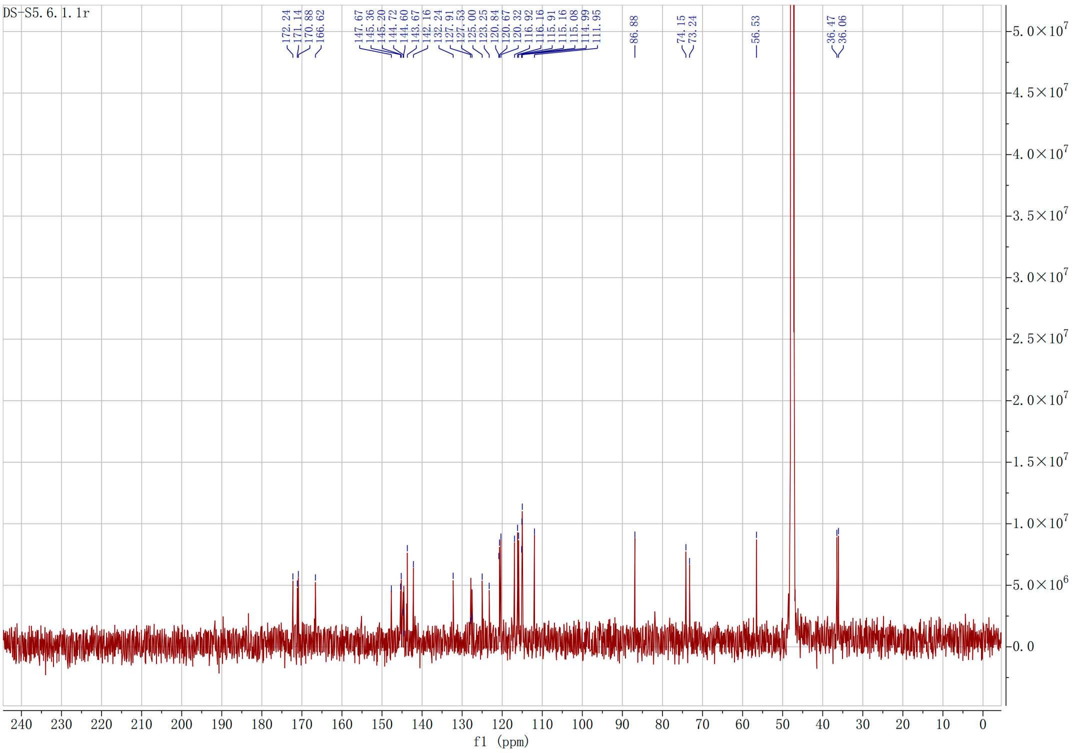

Figure S2. ^{1}H NMR (600 MHz, CD_3OD) spectrum of salvianolic acid B

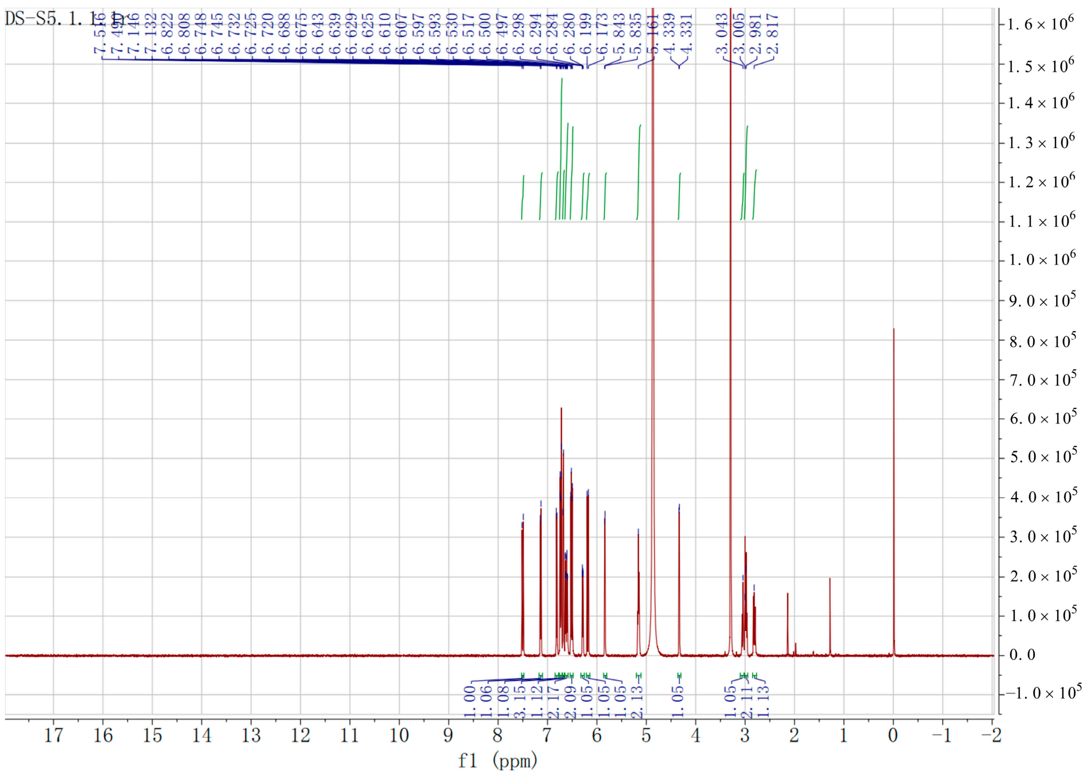

Figure S3. MS spectrum of salvianolic acid B (positive ion mode).

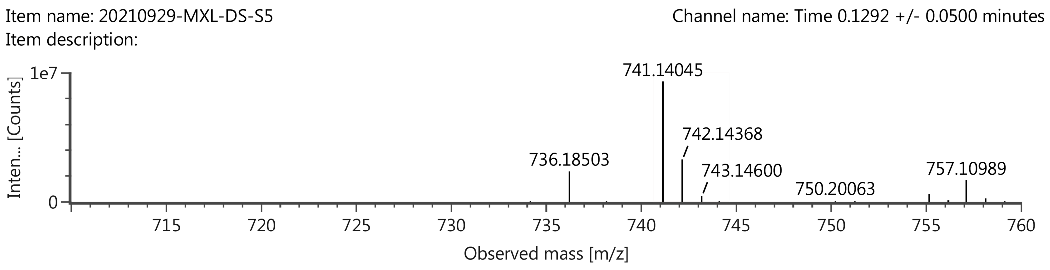

Figure S4. IC_{50} plot of salvianolic acid B and its chemical structure.

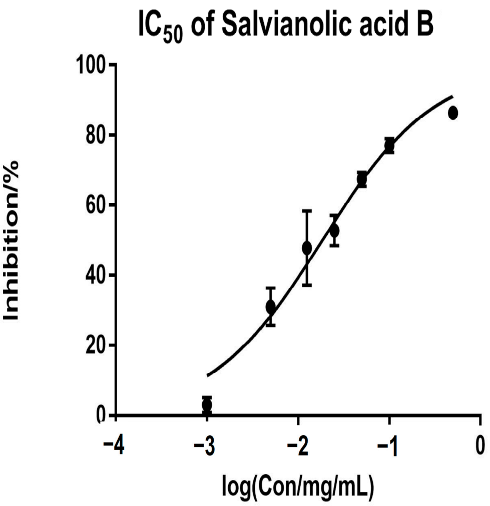

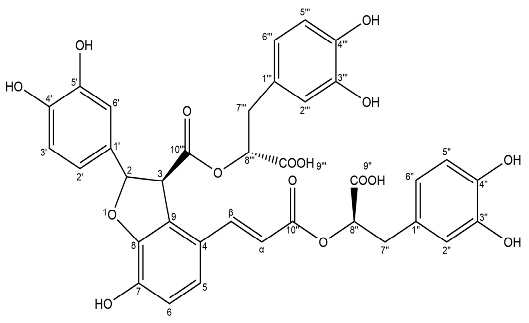

Salvianolic acid B

Figure S5. The authentication letter of crude drug sample.

The authentication letter of crude drug sample

The plant (*Salvia miltiorrhiza* Bunge) was collected from the fields of Wansheng Agricultural Company of Zhongjiang, it was deposited in the Chengdu Institute of Biology, Chinese Academy of Sciences, and its voucher specimen was 2019-07.

Dr. ZHANG Yong-Mei

Herbarium,
Chengdu Institute of Biology,
Chinese Academy of Sciences

Reference

[1] Sun, Y.; Zhu, H.; Wang, J.; Liu, Z.; Bi, J., Isolation and purification of salvianolic acid A and salvianolic acid B from *Salvia miltiorrhiza* by high-speed counter-current chromatography and comparison of their antioxidant activity. *J. Chromatogr. B: Anal. Technol. Biomed. Life Sci.* **2009,** *877,* 733-737.
